# Prediction models developed using artificial intelligence: similar predictive performances with highly varying predictions for individuals – an illustration in deep vein thrombosis

**DOI:** 10.1186/s41512-025-00216-5

**Published:** 2026-01-08

**Authors:** Maerziya Yusufujiang, Constanza L Andaur Navarro, Johanna AA Damen, Toshihiko Takada, Geert-Jan Geersing, Lotty Hooft, Ewoud Schuit, Karel GM Moons, Valentijn MT de Jong, Maarten van Smeden

**Affiliations:** 1 Julius Center for Health Sciences and Primary Care, University Medical Center Utrecht, Utrecht University, Utrecht, The Netherlands; 2 Department of General Medicine, Shirakawa Satellite for Teaching and Research (STAR), Fukushima Medical University, Fukushima, Japan; 3 Department of General Practice & Nursing Sciences, University Medical Center Utrecht, Utrecht University, Utrecht, The Netherlands

**Keywords:** Prediction model, Artificial intelligence, Discrimination, Decision making, Stability of predictions

## Abstract

**Objectives:**

The rise in popularity and off-the-shelf availability of machine learning (ML) and AI-based methodology to develop new prediction models provides developers with ample choices to compare and select the best performing model out of many possible models. Many studies have shown that such comparisons on any particular dataset, the difference in performance between models developed using different techniques (e.g. logistic regression, vs. random forest or neural networks) can often be small, especially when looking at crude performance measures such as the area under the ROC curve. This may lead to the conclusion that such models are essentially exchangeable, and model selection is arbitrary. However, as we will illustrate using a dataset on deep venous thrombosis, prediction models with similar discriminative performance may nonetheless generate different outcome probability estimates for individual patients and potentially lead to meaningfully different decision making.

**Methods:**

We developed diagnostic prediction models to predict the presence of deep venous thrombosis (DVT) in a large dataset of patients with leg symptoms suspected of having DVT, using five modelling techniques: unpenalized logistic regression (ULR), ridge logistic regression (RLR), random forests (RF), support vector machine (SVM) and neural network (NN). Age, sex, d-dimer, history of DVT, diagnosis alternative to DVT, and having cancer were used as a fixed set of predictors. Model performance was evaluated in terms of discrimination, calibration, and stability of individual risk prediction for a set of patients across the models.

**Results:**

Of the 6,087 suspected patients, 1,146 (19%) were diagnosed with DVT based on leg ultrasound (reference test). Three prediction models (ULR, RLR, NN) had similar discrimination with AUCs point estimates of 0.84. However, the 6087 individuals’ estimated probabilities of DVT varied substantially across the five different modelling techniques, highlighting differences in prediction stability. Notably, the RF model tended to overestimate individual risks, while the SVM model tended to underestimate them compared to the other models. While the estimated probabilities were more similar for ULR, RLR and NN, classification measures (sensitivity, specificity, positive and negative predictive value) did differ because of differences in estimated probabilities of individuals near the risk threshold, illustrating that differences, even when relatively small, could potentially lead to different clinical decisions.

**Conclusions:**

Prediction models developed with different modeling techniques yielded very different individuals’ outcome probabilities, even though the models had similar discriminative performance in this low-dimensional setting. Part of this variation can be explained by differences in calibration but also from modelling choices as estimated risks also differed for modelling techniques with similar calibration performance. Hence, our findings highlight the impact of the choice of modelling techniques on model performance, individual estimated probabilities and consequently the impact of that choice on risk-based clinical decision making.

**Supplementary Information:**

The online version contains supplementary material available at 10.1186/s41512-025-00216-5.

## Introduction

Healthcare professionals regularly combine multiple pieces of information to estimate an individual’s probability or risk of having an outcome (diagnosis) or developing an outcome in the future (prognosis) [1–3]. Clinical prediction models (CPMs) are the formal combination of multiple variables (or predictors) to estimate this probability. Traditionally, regression models such as logistic regression and cox regression have often been used to estimate these individual’s estimated probabilities. Machine learning (ML) and artificial intelligence (AI) have become increasingly popular in prediction modelling studies [4].

Several studies have suggested that, in low-dimensional settings, where the number of predictors is relatively small compared to the number of observations, prediction models based on ML and AI can achieve better performance than regression-based models, particularly in terms of discrimination measures such as the area under the receiver operating characteristic curve (AUC), but these differences are often small [5–8]. Evaluation of prediction models typically focuses on the model’s ability to separate participants with and without a particular outcome based on the model’s estimated probabilities (discrimination) [9, 10]. CPMs estimate outcome risks based on measured associations between predictors and outcomes, rather than true individual risks. Nevertheless, when such models are used to support probability-based clinical decision-making, it is important that the estimated probabilities are discriminative, well-calibrated, and stable, since unreliable estimates can result in inconsistent guidance for individual patients [11].

Model instability refers to the variability in predictions that arises from small changes in the data or modelling approach. Riley et al. have highlighted the importance of assessing model stability, particularly during internal validation, to understand how predictions might vary across different samples or modelling techniques [12, 13]. Beyond this, it is also essential to distinguish between different sources of uncertainty in prediction models: aleatoric uncertainty, which stems from inherent randomness in the data (e.g., measurement noise), and epistemic uncertainty, which arises due to limitation in model specification (e.g., choice of model or insufficient data) [14]. Differences in modelling approaches—such as logistic regression versus random forests—may not only reflect different assumptions, but also different ways of capturing or propagating these uncertainties.

In clinical practice, prediction models are not only used to estimate probabilities but also to make classification decisions—such as whether a patient is likely to have deep venous thrombosis (DVT)—by applying a predefined probability threshold [15]. The choice of such a threshold directly affects classification performance measures such as sensitivity, specificity, and positive and negative predictive values of the model for that threshold. Even when different models show similar discriminative ability (e.g., comparable AUCs), differences in the distribution of estimated probabilities can lead to varying classification results even when the same threshold is applied. This is particularly important when clinical decision making is based on this classification, e.g., in a situation where the clinical priority is to avoid false negatives, meaning a low threshold is chosen to maximize sensitivity. Therefore, knowing individual risk estimates across different models and how these estimates affect patient classifications when a probability threshold is applied is essential for evaluating their utility in real-world settings.

The objective of this study is to illustrate the variability between individuals’ probabilities when estimated with different modelling methods, including unpenalized logistic regression (ULR), ridge logistic regression (RLR), random forests (RF), support vector machine (SVM) and neural network (NN), in a setting where the models showed similar discrimination performance.

## Methods

### Data sources

We used data from 8 of the 13 prospective diagnostic studies previously collected for an individual participant data meta-analysis (IPDMA) of patients suspected of deep vein thrombosis (DVT) [16]. Further details on the construction of the dataset can be found elsewhere [16]. In the IPDMA, missing data were imputed. For our illustration, we only used the first imputation set for all models to avoid increased variation in the predictions due to variations in the impact of missing data handling.

### Predictors and outcome

We selected a-priori six predictors: age (continuous), sex (categorical, male/female), d-dimer (categorical, yes/no), previous history of DVT (categorical, yes/no), alternative diagnosis as likely as or more likely than DVT (categorical, yes/no), and active malignancy (categorical, yes/no) based on previously developed prediction models for DVT [17, 18]. No data-driven predictor selection was performed, meaning all six predictors were forced in each of the five models. DVT, the outcome of interest, was defined as a dichotomous outcome (present/absent) [17].

### Modelling techniques

We selected the following modelling techniques: unpenalized logistic regression (ULR), ridge logistic regression (RLR), random forest (RF), support vector machine (SVM), and neural network (NN). RF is an ensemble technique consisting of multiple decision trees trained on bootstrapped sub-sets of the full dataset and different initial variables. The model outputs predicted probabilities for each class, which can then be converted into classifications using a chosen threshold [19, 20]. SVM aims to find the best line that separates the outcome groups — for example, patients with and without DVT. It tries to create the widest possible gap between the two groups, which helps it make more reliable predictions on new patients [21]. NN consists of layers of simple connected units. They learn patterns in the data by adjusting the strength of these connections during training, allowing them to capture complex patterns and make predictions. Last, we applied ULR and RLR, which regress the outcome on the predictor values, using the logistic link function. ULR optimizes the binomial likelihood, whereas RLR optimizes the ridge penalized binomial likelihood.

We emphasize that our aim was not to identify the best-performing model for clinical use, but to illustrate how different modeling approaches can yield varying individual predictions.

### Data analysis

We used the R meta-package “caret” (Classification And Regression Training, https://cran.r-project.org/web/packages/caret/index.html) version 6.0.89 to obtain a series of models (Table S1) based on the common set of predictors (above).

### Model development

We developed five models. For ULR and RLR, predictors were entered as linear terms without interactions. In contrast, the RF, SVM, and NN models can capture non-linear relationships and interactions automatically as part of their standard modeling approach.

We performed a 10-fold cross-validation for tuning algorithm-specific parameters (see Table S1) to optimize the AUC.

For RF, we implemented the model using the RandomForest package, which applies a majority vote. For majority vote the individual probability equals the number of trees that indicate outcome presence, i.e., if we have 500 trees in an RF model, and 400 of them indicate the present of the outcome, the probability would be 80%. We used a grid search to tune mtry (the number of variables randomly sampled at each split), and fixed ntree (the number of trees) at 1000. All other parameters were kept at their default settings. Details of the tuning procedure are provided in Table S2.

For SVM, we used the kernlab [22] package, which implements support vector machines using the optimizers from the libsvm software [23]. The probabilities are obtained after a second regression model has been trained on the SVM outputs.

For NN, we used the nnet package through caret to build a model with one hidden layer. Neural networks inherently output probabilities through their activation functions, typically using a sigmoid function (S-shape) in the output layer for binary classification. When no hidden layer is used, a neural network is equivalent to logistic regression. With a hidden layer, it becomes a more flexible model that can capture non-linear relationships.

### Sample size

We used the R package pmsampsize [24] to calculate the sample size required to develop a new prediction model based on logistic regression. One is required to input the overall fraction of participants expected to develop DVT (18%), the number of candidate predictor parameters (*n* = 6) and the anticipated c-statistic (0.81). This c-statistic was informed by previous work on clinical prediction models for suspected DVT that reported similar levels of discriminative performance [25]. The minimum sample size required was at least 266 participants with 48 events, and 7.98 events per candidate predictor parameter (EPP) while considering a shrinkage factor of 0.90 and R^2^ Cox-Snell (R^2^cs) of 0.18.

### Assessment of model performance and classification

We applied each model to the dataset to obtain estimated probabilities for each individual in the dataset. We determined the models’ discriminative performance by calculating the model’s c-index (AUC). A perfect model would have an AUC equal to 1. We also assessed models’ calibration graphically by calibration plots [11]. If a model is well calibrated, there is perfect agreement between model-estimated risks (x-axis) and observed outcome frequencies (y-axis), resulting in a diagonal line in the plot.

In addition, we assessed patient classification with sensitivity, specificity, positive predictive value (PPV), and negative predictive value (NPV). While AUC is probability threshold-independent, classification measures depend on the threshold applied to the predicted model probabilities. In this study, we used a fixed threshold of 0.02 to classify patients as having or not having DVT. This low threshold was explicitly chosen to directly reflect the clinical importance of prioritizing sensitivity, given the potentially life-threatening consequences of a missed DVT diagnosis [17, 26, 27]. This approach aligns with clinical decision-making in high-risk scenarios and allows for meaningful comparison of model predictions under a uniform classification rule.

Estimated probabilities for the same individuals were compared across different modeling techniques using a scatter plot. Additionally, for a random selection of 10 individuals in the dataset, we illustrated the estimated probabilities across the modeling techniques. To explore potential differences in estimated probabilities, we further examined the strength of the association of the six predictors with the outcome across the different modeling techniques using model coefficients (for ULR and RLR) and variable importance measures (for RF, SVM, and NN). Additionally, we used the iml package to generate Individual Conditional Expectation (ICE) plots, which helped visualize how changes in each predictor affected the estimated probabilities for individual patients.

All statistical analyses were performed in R version 4.0.3 [28] with the crossTable, rms, pROC, ggplot, and caret packages.

## Results

The dataset included 6,087 patients of whom 1,146 (19%) were confirmed cases of DVT presence. The mean age was 59 (SD ± 17) years for all participants, of whom 3760 (62%) were female. Further details on the study participants can be found in the original publication [16] and in Table 1.

**Table 1:** Characteristics of the study population in terms of predictors, overall, and stratified by outcome presence

	Total, *n* = 6087 (100)	DVT, *n* = 1146 (19)	No DVT, *n* = 4941 (81)
Age, mean (SD)	59 (± 17)	61 (± 17)	59 (± 17)
Sex, count (%)			
Female	3760 (62)	602 (53)	3158 (64)
Male	2337 (38)	544 (48)	1783 (36)
D-dimer, count (%)			
No	3114 (51)	99 (9)	3015 (61)
Yes	2973 (49)	1047 (91)	1926 (39)
Cancer, count (%)			
No	5486 (90)	918 (80)	4568 (93)
Yes	601 (10)	228 (20)	373(8)
Previous history of DVT, count (%)			
No	5747 (94)	1055 (92)	4692 (95)
Yes	340 (6)	91 (8)	249 (5)
Alternative diagnosis, count (%)			
No	3145 (52)	919 (80)	2226 (45)
Yes	2942 (48)	227 (20)	2715 (55)

### Tuning parameters

The values for hyperparameters of the ML models after tuning are presented in Table S2.

### Discrimination

The five models achieved AUCs ranging from 0.81 to 0.84, with the RF model showing the lowest AUC (0.81, 95% CI 0.80–0.82), SVM model also showing relatively low AUC (0.82, 95%CI 0.81–0.83) and the ULR (0.84, 95% CI 0.83–0.85), RLR (0.84, 95% CI 0.83–0.85), and NN (0.84, 95% CI 0.83–0.85) models showing the highest AUC.

### Calibration

**Fig. 1:**
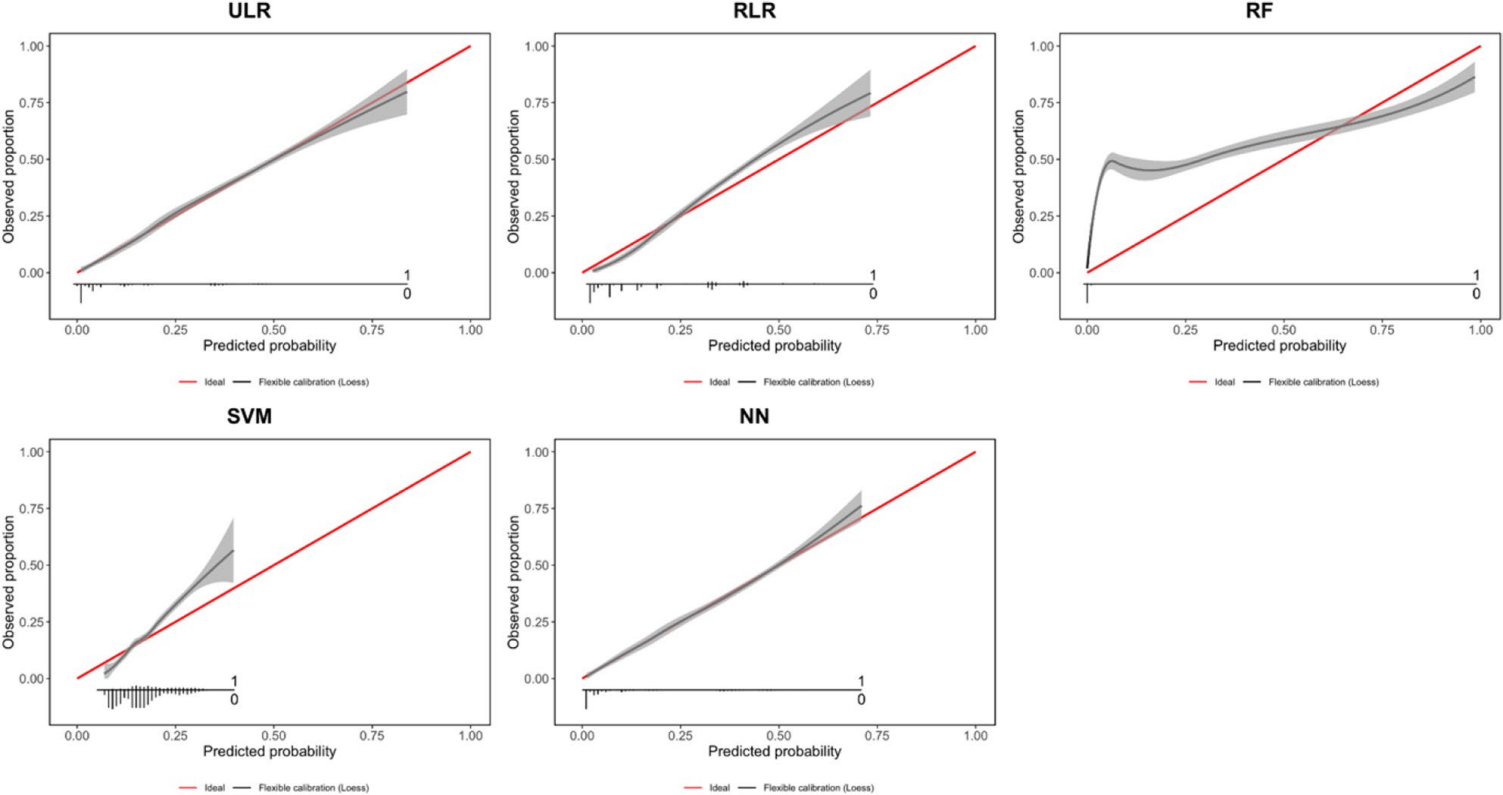
Calibration plot for five models. Each plot illustrates the agreement between the probabilities estimated by the algorithm (x-axis) and the observed proportion of individuals in the dataset with the outcome (y-axis). Perfect calibration is indicated by the red diagonal line. The distribution of individuals with (1) and without (0) the outcome (DVT) is presented at the bottom of each calibration plot. The calibration of the model is indicated by the black line, while the grey area illustrates the 95% confidence interval of this calibration. ULR = unpenalized logistic regression, RLR = ridge logistic regression, RF = random forests, SVM = support vector machine and NN = neural network

The calibration plots are shown in Fig. 1 and calibration statistics for five models are provided in Table S4. The ULR and NN models showed good calibration across the range of predicted probabilities, while RLR slightly underestimated higher risks. In contrast, RF and SVM demonstrated clear miscalibration, with RF overestimating and SVM underestimating predicted probabilities in parts of the risk range.

These differences indicate that, despite similar discrimination, models varied in how accurately their estimated probabilities reflected observed outcomes. Such variation in calibration helps explain the differences in individual predicted risks observed across models, which we investigated in the next subsection.

### Classification

Table 2 summarizes the classification performance of the five fitted models. All models had similar AUCs, but sensitivity, specificity, and predictive values varied across models. This variation partially reflects differences in calibration: RF and SVM showed poorer calibration and therefore diverged in classification performance. However, ULR and NN, and to some extent RLR, showed good calibration, and still differences in classification measures were observed because of differences in estimated probabilities of individuals near the risk threshold across models. In contrast, RF provided a more balanced sensitivity (0.81) and specificity (0.81), resulting in the highest PPV (0.50) among all models. The neural network also performed relatively well, with sensitivity of 0.82 and specificity of 0.75.

**Table 2:** Performance measures

Model	Performance measures				
	AUC (95% CI)	Sensitivity (95% CI)	Specificity (95% CI)	PPV (95% CI)	NPV (95% CI)
Unpenalized logistic regression	0.84 (0.83-0.85)	0.98 (0.98-0.99)	0.31 (0.30-0.33)	0.25 (0.24-0.26)	0.98 (0.98-0.99)
Ridge logistic regression	0.84 (0.83-0.85)	1.00	0	0.19 (0.18-0.19)	NaN
Random Forest	0.81 (0.80-0.82)	0.81 (0.79-0.83)	0.81 (0.80-0.82)	0.50 (0.47-0.52)	0.94 (0.94-0.95)
Support Vector machine	0.82 (0.81-0.83)	1.00	0	0.19 (0.18-0.19)	NaN
Neural Network	0.84 (0.83-0.85)	0.82 (0.79-0.84)	0.75 (0.73-0.76)	0.43 (0.41-0.45)	0.95 (0.94-0.95)

*CI* Confidence interval. The 95% confidence intervals for AUC were calculated using the DeLong method and for classification measures using the Wilson score interval. *PPV* Positive predictive value. *NPV* Negative predictive value. *NaN* Not a Number

### Individual estimated probabilities

**Fig. 2:**
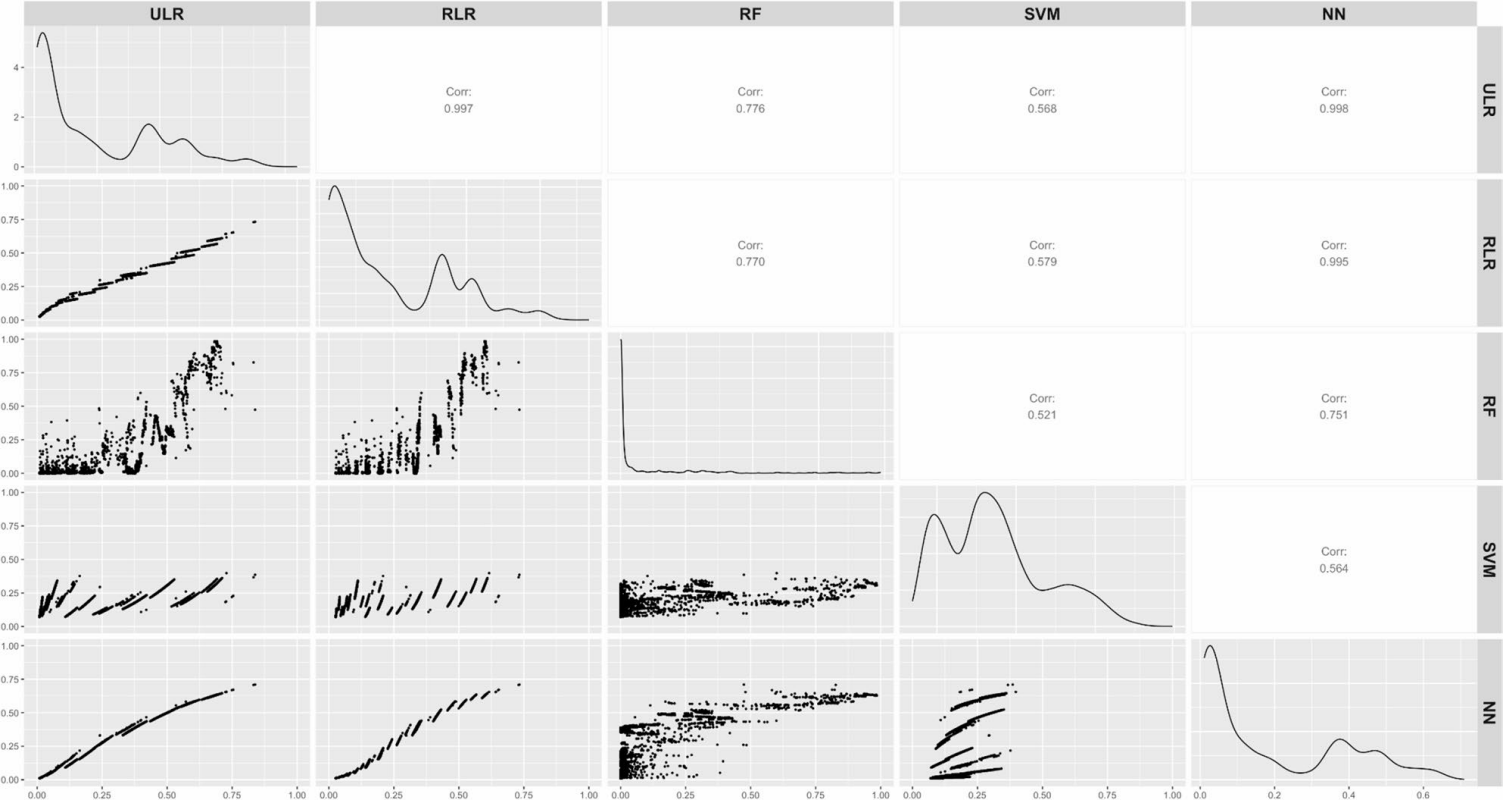
Pairwise scatterplot matrix comparing estimated probabilities from five models. ULR = unpenalized logistic regression, RLR = ridge logistic regression, RF = random forests, SVM = support vector machine and NN = neural network. Each panel below the diagonal displays scatter plots of estimated probabilities between two models, where plots showing a diagonal line would indicate perfect agreement of estimated probabilities between models. Panels on the diagonal show the distribution (density) of estimated probabilities for each individual model. Panels above the diagonal present the Pearson correlation coefficients (indicated as Corr.) quantifying the similarity in estimated probabilities between model pairs. A correlation (Corr.) value close to 1 indicates strong agreement between two models, while lower values reflect divergence in their predictions

As can be seen from the calibration plots in Fig. 1 and also from the plots on the diagonal of Fig. 2, the range of estimated probabilities substantially differed between modelling techniques. While RF provided a broad range of probabilities (from 0 to 1), NN showed a narrower range (from 0 to 0.7). Likewise, the estimated probabilities are not equally distributed across the range of probabilities for the different modeling techniques, although most individual probabilities were found at the lower end of the distribution. For example, most individual probabilities provided by the RF model were close to 0.

**Fig. 3:**
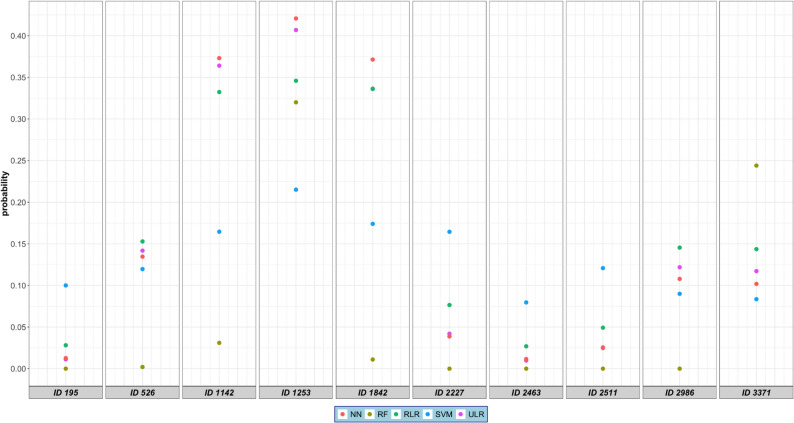
Comparison of estimated probabilities across five different modeling methods for ten random individuals. ULR = unpenalized logistic regression, RLR = ridge logistic regression, RF = random forests, SVM = support vector machine and NN = neural network

Figure 3 and Table S3 show a comparison of the different estimated probabilities for ten random individuals, indicating that estimated probabilities can differ substantially across methods within the same individual. For example, for individual with ID 195, RLR predicted a probability of 0.028 of DVT being present, which is above the threshold (0.02) commonly used for diagnosing DVT in practice, and NN and ULR respectively estimated probabilities of 0.012 and 0.011, which are below the threshold. Even larger differences were observed when the models with a lower estimate for the AUC were considered. For the same individual, RF estimated a probability of 0.00, and SVM a probability of 0.10. A similar pattern was observed for ID 2463. Further, the individual with ID 1142 had a 0.36 probability based on the ULR model, while the models based on the other methods provided estimated probabilities from 0.03 (RF) to 0.37 (NN) for that same individual. Although these differences are large, they would not lead to different clinical decisions based on the threshold of 0.02.

### Predictors

We generated the Individual Conditional Expectation (ICE) plots (see Figures S1-S6) to assess the contribution of individual predictors. The plots revealed that d-dimer value and alternative diagnostic were the most important predictors across all models. However, differences were found for the RF and SVM models as compared to ULR, RLR, and NN models.

## Discussion

We showed that prediction models developed using different modelling techniques can yield comparable discrimination in a low-dimensional setting but differ substantially in calibration, which can partly explain why individual estimated probabilities varied across models. Even though two models may rank patients similarly in terms of estimated probabilities (reflected in the same high discrimination of both models), they may provide very different absolute estimated probabilities for the same individual. This can potentially lead to different clinical decisions for the same patient across modeling techniques. For example, we observed that ULR and NN produced highly correlated probabilities, which frequently but not always led to the same decision at the 2% probability level, while SVM and RF showed weaker correlations and poorer calibration. Once adequate calibration and discrimination are established, examining the stability of individual predicted probabilities across models offers additional insight into those estimates. When well-calibrated models produce similar predictions, this consistency increases confidence in their reliability.

Our findings emphasize the need for careful consideration in individual-level predictions when comparing and choosing models for decision making in daily practice. To evaluate their practical utility, methods such as decision curve analysis and net benefit can be used to assess whether predicted risks lead to improved decision-making across relevant thresholds [29]. Though in practice, the development and validation of a single, well-suited model is preferable; our use of multiple models here served only to demonstrate the variability in individual predictions.

These differences in estimated probabilities reflect variability in predictions that can arise from the modelling choices themselves. Riley et al. have highlighted the importance of assessing such variability to understand how well models generalize [12, 13]. Our study extends this idea by showing that predictions can vary not only within one modelling method, but also between different modelling techniques. This variation also relates to two types of uncertainty in prediction modelling: aleatoric uncertainty, which comes from randomness in the data, and epistemic uncertainty, which stems from modelling choices [14]. Different modelling approaches handle epistemic uncertainty in different ways, which helps explain why predictions can vary even when models have similar AUCs.

In medical practice, decisions often rely on applying a decision threshold to the model’s estimated probability. Performance measures such as sensitivity, specificity, positive predictive value (PPV), and negative predictive value (NPV) can be strongly influenced by how individual risks are distributed around that threshold. In our study, even models with similar discrimination and good calibration (ULR, RLR, and NN) produced different classification results. This highlights the importance of examining the full distribution of predicted risks and understanding how these distributions interact with clinically meaningful probability thresholds. Evaluating only threshold-independent metrics can overlook important practical implications for patient care. Accurately predicting the probability of DVT presence in suspected patients can help decide on their further diagnosis management and subsequent treatments.

### Comparison to previous research

A previous study showed that prognostic predictions for individual probabilities of cardiovascular disease (CVD) varied between and within different types of machine learning and regression models. In this study, a patient with a probability of 9.5–10.5% predicted by QRISK3, (a well-stablished CVD risk score) had a probability of 2.9–9.2% in a random forest and 2.4–7.2% in a neural network [30]. A study evaluating the performance of three different existing prediction models based on regression techniques for cardiovascular disease concluded that their application may result in considerable misclassification for individuals with the highest probabilities [31]. 

### Strengths and limitations of this study

This analysis included individual participant data from more than 6,000 participants with suspected DVT, of whom 1146 were diagnosed with the outcome of DVT presence. One strength of our approach is that it shows the impact of different models yielding estimated probabilities in a real-life scenario where a probability-based classification threshold (0.02) is typically used, and missing a DVT diagnosis can be potentially life-threatening due to clot progression to pulmonary embolism should the initial DVT be left untreated. Another strength is that we applied consistent model development procedures to enable a fair comparison of predictive performance across five modelling approaches, including both traditional regression and machine learning techniques. Each model was properly tuned using internal resampling (e.g., selecting the regularization parameter for ridge regression, the number of trees for RF, and the cost parameter for SVM).

This study also has some limitations. We used the caret package for model training and tuning across all methods. While caret provides a convenient and unified framework, recent research has shown that certain tuning methods, especially the popular one-standard-error rule in cross-validation, can lead to poor calibration of prediction models, even when discrimination is good [32].

Finally, this study was designed as an illustrative comparison rather than a model-development study. We do not recommend fitting multiple models for every medical prediction task. Choosing a modelling approach should be guided by the clinical context and the specific requirements of the task, for example, by considering interpretability, model performance (e.g., discrimination, calibration), data structure, and how well the model supports decision-making in situations where certain errors (e.g., false negatives) carry serious consequences (such as in DVT or breast cancer). Factors like model interpretability, data structure, and intended clinical use should guide this choice. Understanding how modeling decisions affect both individual predictions and performance metrics can help researchers align model development with clinical priorities and practical constraints.

### Implications for researchers and future research

Our intention in including models with poor calibration (e.g. RF and SVM) was not to advocate for their clinical use, but to highlight that calibration can differ substantially between modeling approaches, even when discrimination (AUC) is similar. This highlights that reliance on AUC alone provides an incomplete assessment of model performance, as it does not reflect how accurately predicted probabilities correspond to observed outcomes.

Systematic reviews have found that calibration is assessed far less often compared to discrimination, irrespective of whether models were built using ML, AI or statistical techniques [9, 10]. The lack of calibration limits the use of clinical prediction models and, consequently, several guidelines have stressed the need to report calibration alongside discrimination [33]. Moreover, as illustrated by Table S3, prediction intervals for individual risks can differ across methods. Particularly for SVM and RF, prediction intervals were wide for some individuals, indicating large uncertainty in estimated risks for these methods for these patients. Beyond calibration, the stability of predictions across modelling approaches may also offer useful insights into the robustness of model results [13].

As shown in this study, details on the different modeling techniques and parameters used is necessary for critical appraisal. To provide guidance on reporting and critical assessment, the TRIPOD + AI and PROBAST + AI have been recently published [34, 35].

## Conclusion

Even in a low-dimensional setting where different modelling techniques achieve similar discrimination, they can produce markedly different individual risk estimates. These differences arise not only from variation in calibration but also from the modelling choices themselves. These findings highlight that the selection of a modelling technique can meaningfully affect model performance, individual estimated probabilities and the choice on clinical decisions that depend on risk thresholds.

## Supplementary Information

Supplementary Material 1: Table S1. Details on model development [22, 36–38]. Table S2. Tuning of parameters based on repeated cross-validation. Table S3. Risk probabilities and prediction interval for ten random individuals. Table S4. Calibration statistics (95% Confidence Intervals) for five models. Figure S1: ICE Plot of age for the 5 different models. Figure S2. ICE Plot of sex for the 5 different models. Figure S3. ICE Plot of history of previous DVT for the 5 different models. Figure S4. ICE Plot of dichotomized d-dimer value for the 5 different models. Figure S5. ICE Plot of active malignancy for the 5 different models. Figure S6. ICE Plot of alternative diagnosis for the 5 different models.

## Data Availability

The DVT data that support the findings of this study are not publicly available, according to the conditions determined by the authors of the DVT studies, but are available on reasonable request from GJG by e-mail.Analytical code is available via repository [https://github.com/Maerziya/EstimatingRisks].

## Abbreviations

DVT: Deep venous thrombosis
ULR: Unpenalized logistic regression
RLR: Ridge logistic regression
RF: Random forests
SVM: Support vector machine
NN: Neural network
IPDMA: Individual participant data meta-analysis
