# Supplementary material for: Prediction models developed using artificial intelligence: similar predictive performances with highly varying predictions for individuals – an illustration in deep vein thrombosis
Source: Diagn Progn Res. 2026 Jan 8;10:1. doi: 10.1186/s41512-025-00216-5 (PMC12784591; doi:10.1186/s41512-025-00216-5)

## **Supplementary material**

**Table S1**. Details on model development

| Model | Independent R packages | Methods in “caret” package | Tuning parameters |
| --- | --- | --- | --- |
| Unpenalized logistic regression (ULR) | glm(22) | glm* | none |
| Ridge logistic regression (RLR) | glmnet(22) | glmnet* | lambda |
| Random Forest (RF) | randomForest(23) | rf | mtry, ntree |
| Support vector machine with linear kernel (SVM) | kernlab(24) | svmLinear | C |
| Neural Network (NN) | nnet(25) | nnet | size, decay |

*glm and glmnet used a binomial family and logit link function

Table S2. Tuning of parameters based on repeated cross-validation.

| Model | Tuning parameters | Value |
| --- | --- | --- |
| Unpenalized logistic regression | Not applicable | Not applicable |
| Ridge logistic regression | lambda | 0.001 to 0.1 |
| Random Forest | mtry | 3 |
|  | ntree | 1000 |
|  | nodesize | 1 |
|  | maxnodes | NULL (max possible) |
|  | strata | NULL |
| Support vector machine with linear kernel | C | 1 |
| Neural Network | size | 1 |
|  | decay | 0.1 |

Table S3. Risk probabilities and prediction interval for ten random individuals.

| ID | DVT Status | ULR | RLR | RF | SVM | NN |
| --- | --- | --- | --- | --- | --- | --- |
|  | 0/1 |  |  |  |  |  |
| 195 | 0 | 0.0112 (0.0088–0.0144) | 0.0281 (0.0246–0.0316) | 0.0000 (0.0000–0.0000) | 0.1000 (0.0071–0.2040) | 0.0127  (0.0089–0.0180) |
| 526 | 0 | 0.1418 (0.1195–0.1675) | 0.1529 (0.1349–0.1717) | 0.0020 (0.0000–0.0048) | 0.1196 (0.0427–0.2937) | 0.1346  (0.1109–0.1606) |
| 1142 | 1 | 0.3641 (0.3372–0.3918) | 0.3323 (0.3095–0.3557) | 0.0310 (0.0203–0.0417) | 0.1646 (0.1200–0.4230) | 0.3731  (0.3467–0.4033) |
| 1253 | 1 | 0.4069 (0.3630–0.4523) | 0.3459 (0.3116–0.3797) | 0.3200 (0.2911–0.3489) | 0.2151 (0.1215–0.5009) | 0.4207  (0.3828–0.4636) |
| 1842 | 0 | 0.3364 (0.2835–0.3938) | 0.3360 (0.2917–0.3783) | 0.0110 (0.0045–0.0175) | 0.1740 (0.0907–0.6442) | 0.3714  (0.3087–0.4379) |
| 2227 | 0 | 0.0420 (0.0340–0.0519) | 0.0765 (0.0689–0.0851) | 0.0000 (0.0000–0.0000) | 0.1644 (0.0405–0.2465) | 0.0388  (0.0302–0.0482) |
| 2463 | 0 | 0.0098 (0.0074–0.0129) | 0.0269 (0.0232–0.0309) | 0.0000 (0.0000–0.0000) | 0.0796 (0.0067–0.2032) | 0.0115  (0.0078–0.0169) |
| 2511 | 0 | 0.0247 (0.0172–0.0354) | 0.0492 (0.0385–0.0620) | 0.0000 (0.0000–0.0000) | 0.1208 (0.0197–0.3751) | 0.0255  (0.0172–0.0373) |
| 2986 | 0 | 0.1219 (0.1037–0.1427) | 0.1455 (0.1295–0.1621) | 0.0000 (0.0000–0.0000) | 0.0899 (0.0402–0.2405) | 0.1078  (0.0863–0.1278) |
| 3371 | 1 | 0.1172 (0.0981–0.1394) | 0.1436 (0.1265–0.1614) | 0.2440 (0.2174–0.2706) | 0.0835 (0.0362–0.2406) | 0.1018  (0.0792–0.1238) |

Table S4: Calibration statistics (95% Confidence Intervals) for five models

|  | ULR | RLR | RF | SVM | NN |
| --- | --- | --- | --- | --- | --- |
| Calibration intercept | 0  (-0.07-0.07) | 0  (-0.07-0.07) | 1.80  (1.70-1.91) | 0.12  (0.05-0.18) | 0  (-0.07-0.07) |
| Calibration slope | 1.00  (0.93-1.07) | 1.36  (1.28-1.45) | 0.41  (0.38-0.43) | 1.69  (1.53-1.85) | 1.01  (0.94-1.08) |

ICE plots of 6 predictors for the 5 different models

The x-axis labels “Neg” and “Pos” are outcome labels (whether DVT is absent or present). Each plot shows how predicted probabilities of DVT change when predictor is varied (0 /1 ), stratified by the true outcome (Neg = no DVT, Pos = DVT).

0 and 1 indicate the the predictors status as below:

For the ICE plot for sex, female = 0 and male = 1

For the ICE plot for history of previous DVT, no history of previous DVT = 0 and history of previous DVT = 1

For the ICE plot for dichotomized d-dimer value,D-dimer negative = 0 and D-dimer positive = 1

For the ICE plot for active malignancy, no active malignancy = 0 and active malignancy = 1

For the ICE plot for alternative diagnosis present, no alternative diagnosis present = 0 and alternative diagnosis present = 1 "

Figure S1: ICE Plot of age for the 5 different models.


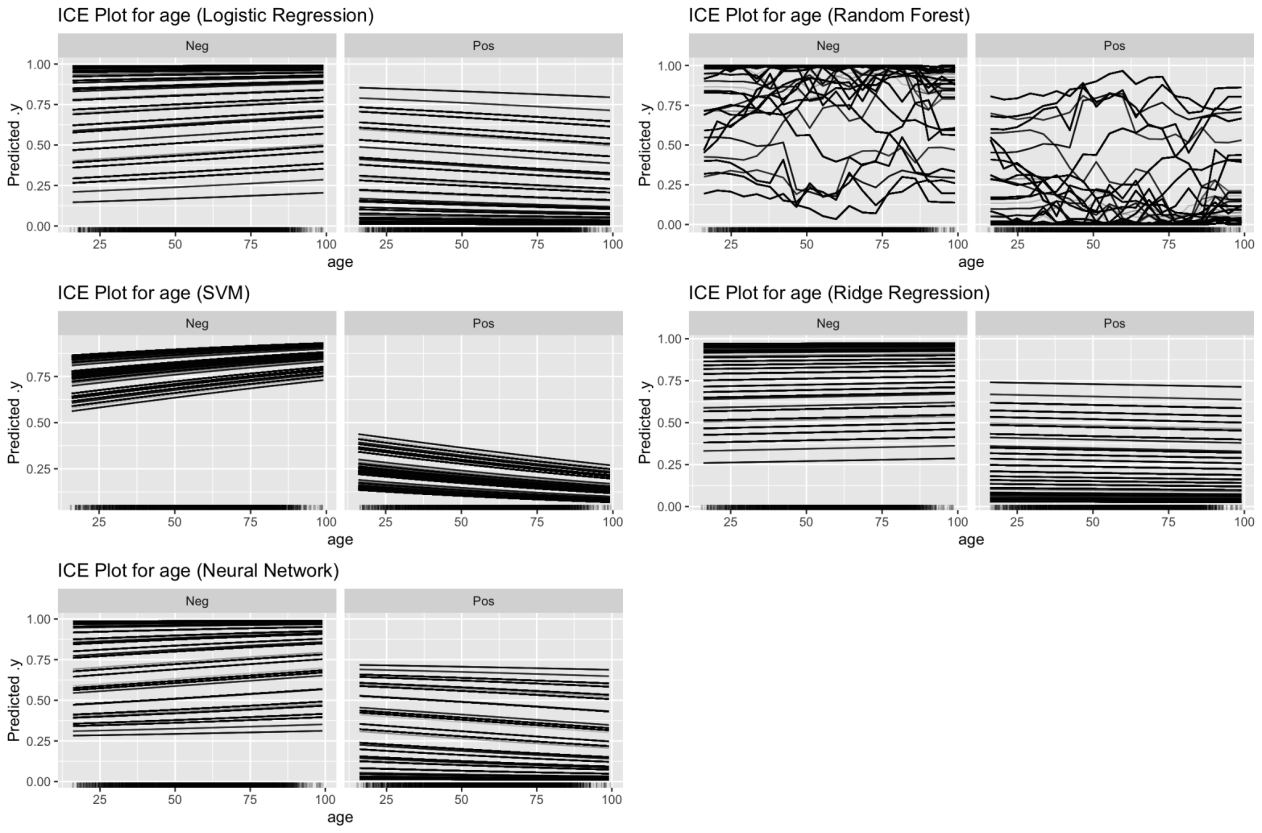


Figure S2: ICE Plot of sex for the 5 different models.


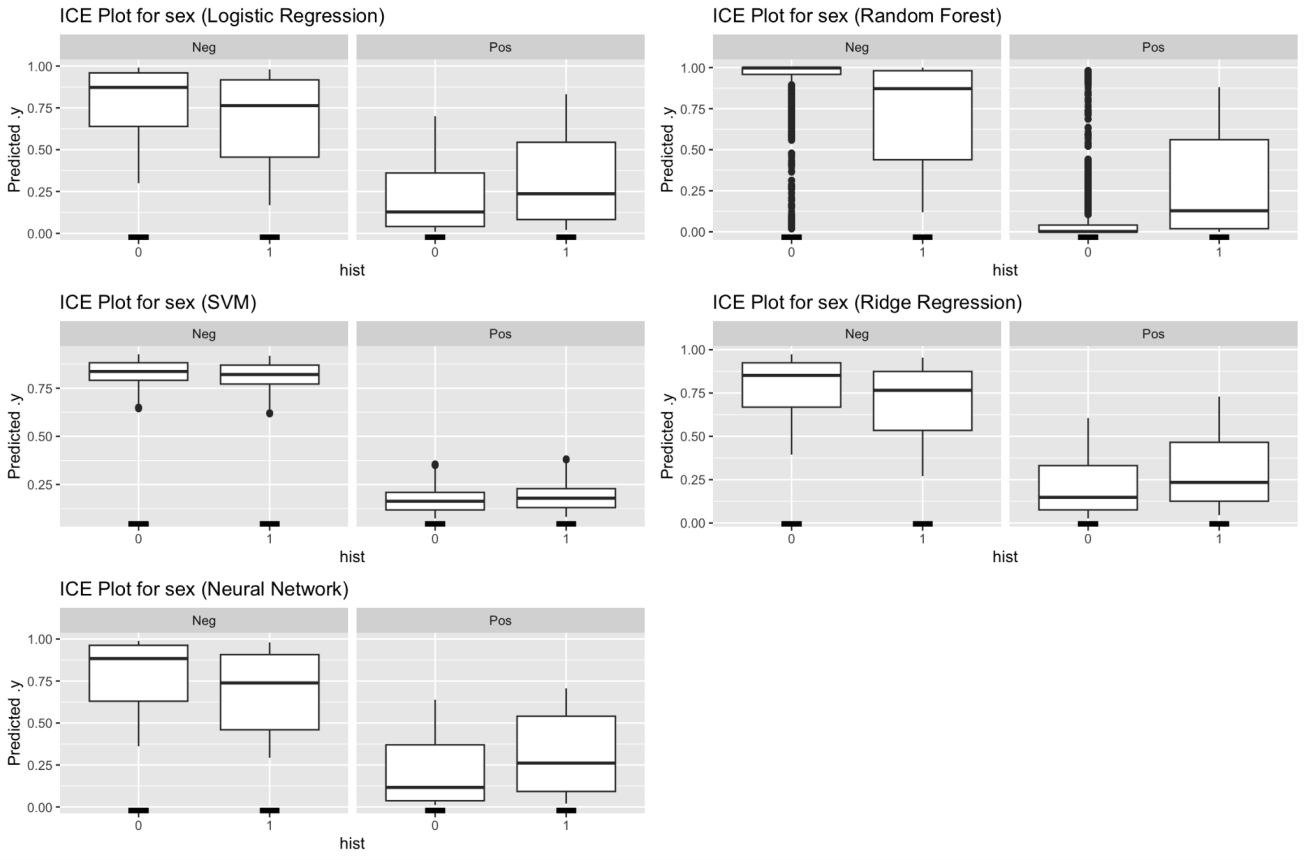


Figure S3: ICE Plot of history of previous DVT for the 5 different models.


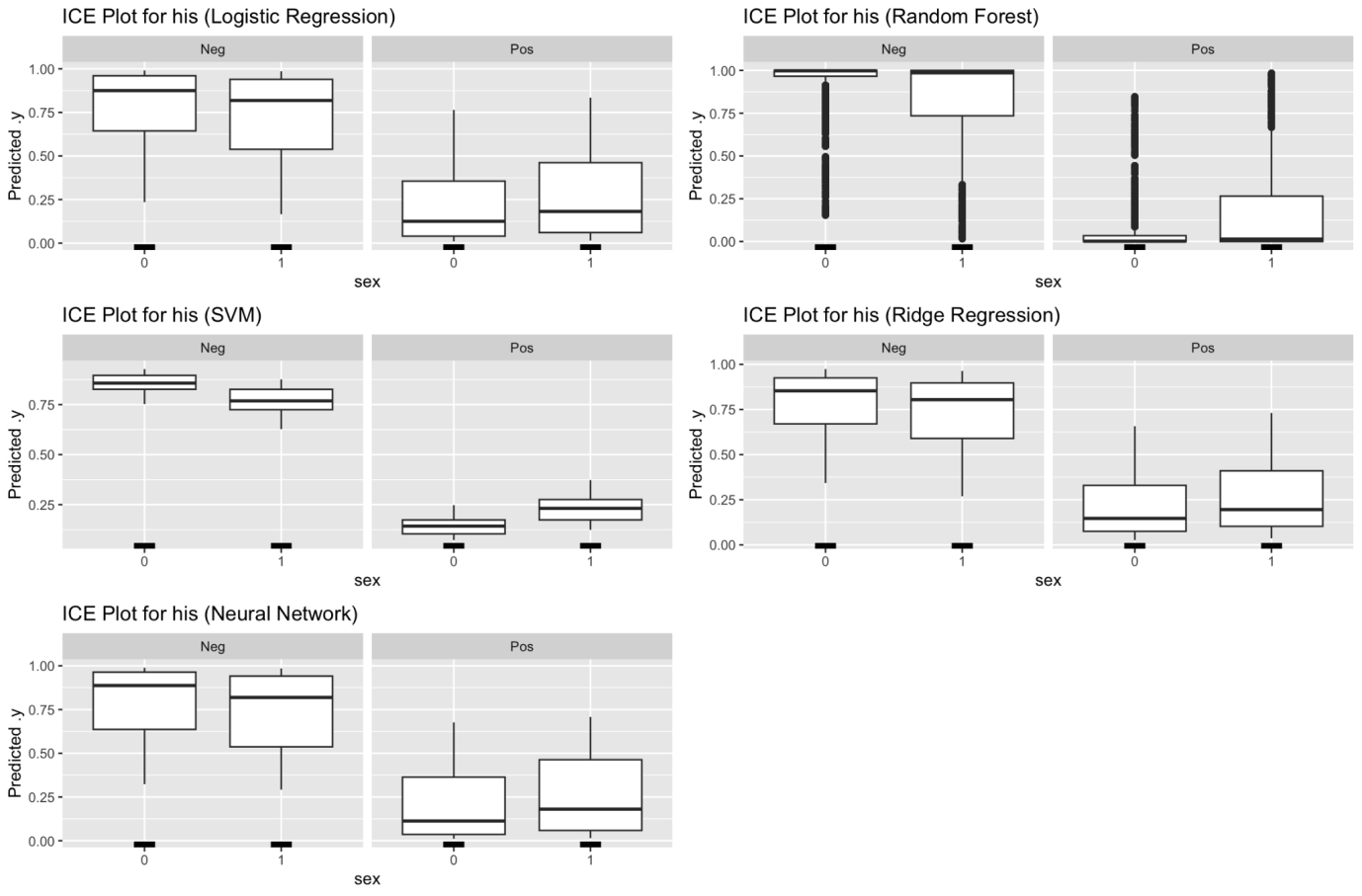


Figure S4: ICE Plot of dichotomized d-dimer value for the 5 different models.


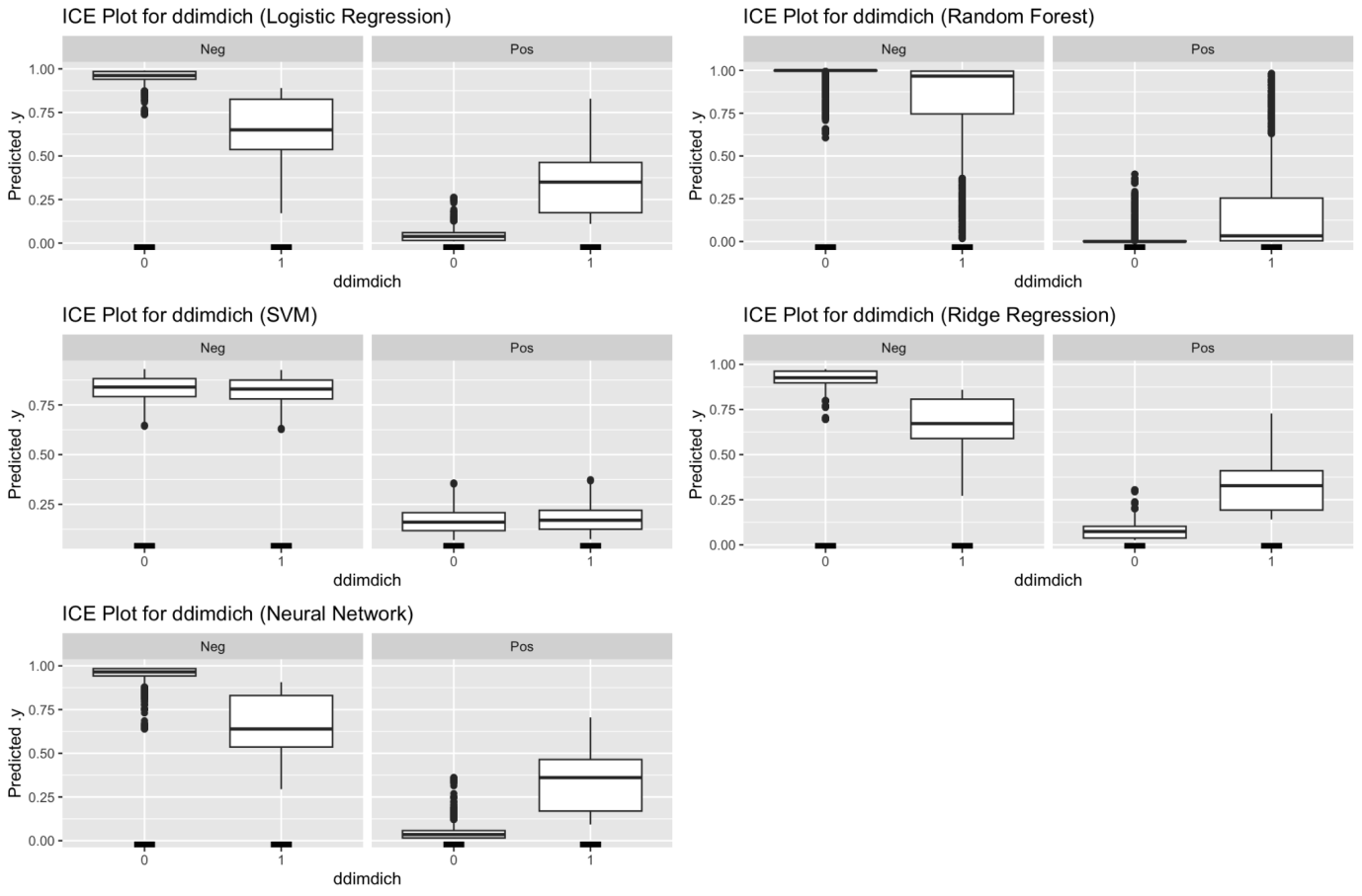


Figure S5: ICE Plot of active malignancy for the 5 different models.


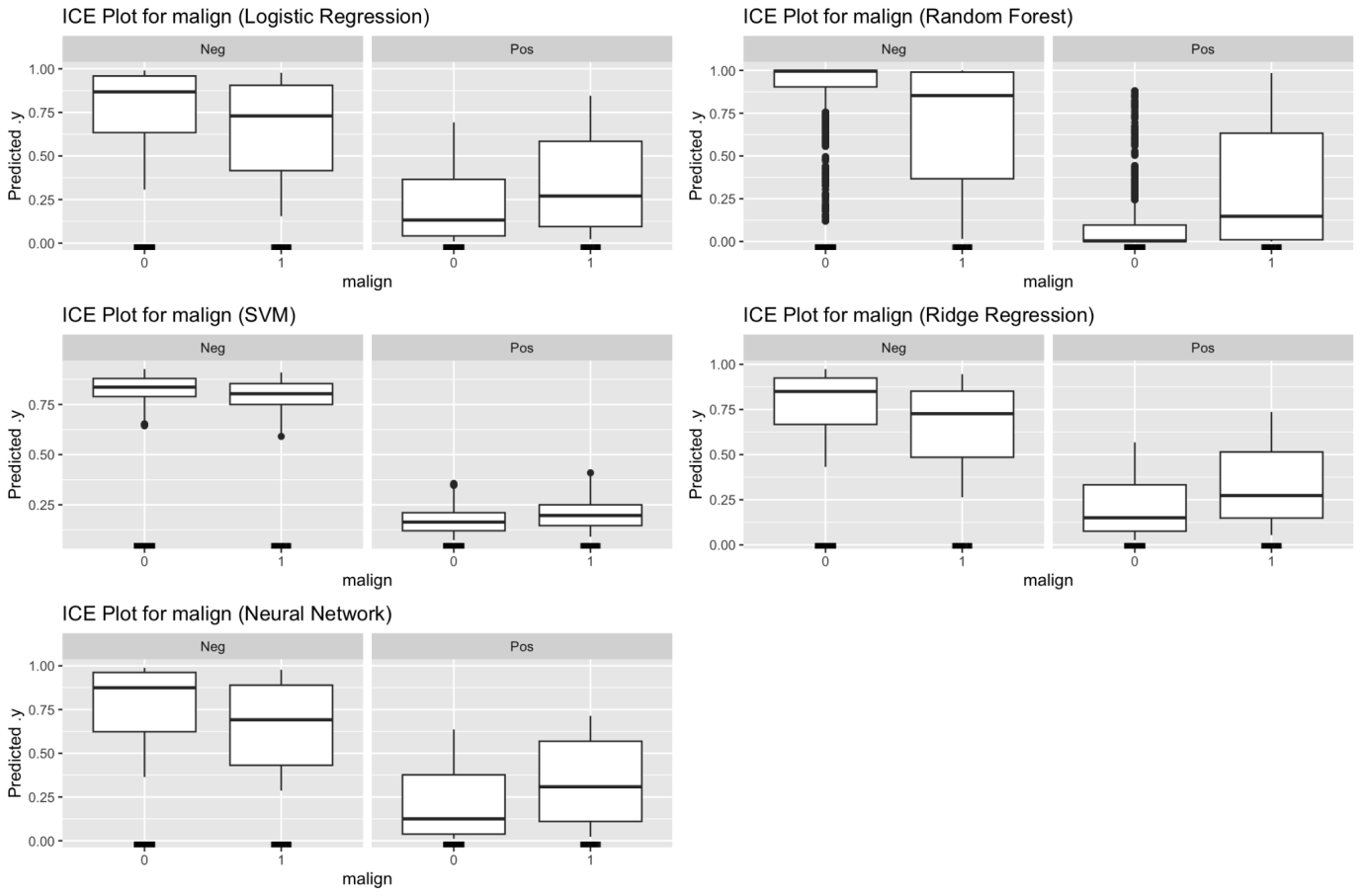


Figure S6: ICE Plot of alternative diagnosis for the 5 different models.


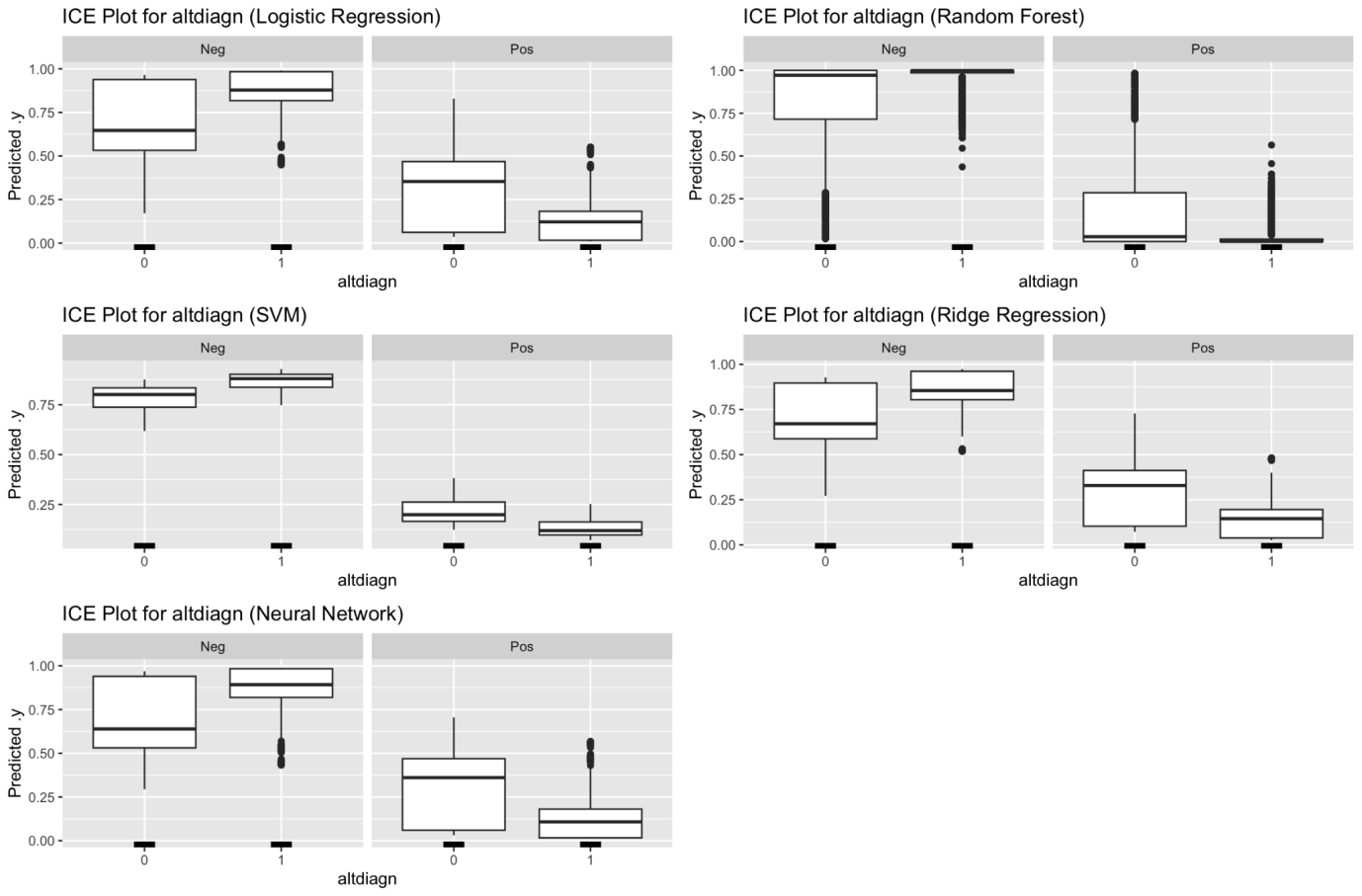

Supplement: Supplementary file 1 — Supplementary Material 1: Table S1. Details on model development [22, 36–38]. Table S2. Tuning of parameters based on repeated cross-validation. Table S3. Risk probabilities and prediction interval for ten random individuals. Table S4. Calibration statistics (95% Confidence Intervals) for five models. Figure S1: ICE Plot of age for the 5 different models. Figure S2. ICE Plot of sex for the 5 different models. Figure S3. ICE Plot of history of previous DVT for the 5 different models. Figure S4. ICE Plot of dichotomized d-dimer value for the 5 different models. Figure S5. ICE Plot of active malignancy for the 5 different models. Figure S6. ICE Plot of alternative diagnosis for the 5 different models. [file 41512_2025_216_MOESM1_ESM.docx]
